# Pathogenic Pseudorabies Virus, China, 2012

**DOI:** 10.3201/eid2001.130531

**Published:** 2014-01

**Authors:** Xiuling Yu, Zhi Zhou, Dongmei Hu, Qian Zhang, Tao Han, Xiaoxia Li, Xiaoxue Gu, Lin Yuan, Shuo Zhang, Baoyue Wang, Ping Qu, Jinhua Liu, Xinyan Zhai, Kegong Tian

**Affiliations:** China Animal Disease Control Center, Beijing, China (X. Yu, Z. Zhou, D. Hu, Q. Zhang, T. Han, X. Li, X. Gu, L. Yuan, S. Zhang, B. Wang, P. Qu, X. Zhai, K. Tian);; China Agricultural University, Beijing (D. Hu, Q. Zhang, J. Liu);; National Research Center for Veterinary Medicine, Luoyang, Henan, China (K.Tian)

**Keywords:** pseudorabies, highly pathogenic PRV, China, viruses, pigs, swine

## Abstract

In 2012, an unprecedented large-scale outbreak of disease in pigs in China caused great economic losses to the swine industry. Isolates from pseudorabies virus epidemics in swine herds were characterized. Evidence confirmed that the pathogenic pseudorabies virus was the etiologic agent of this epidemic.

Pseudorabies virus (PRV), also called Aujeszky disease virus or suid herpesvirus type 1, is a member of the *Alphaherpesvirinae* subfamily within the family *Herpesviridae*. This pathogen has major economic consequences in pig husbandry (*1*–*3*). The PRV genome is a double-stranded linear DNA molecule ≈143 kb long and contains at least 72 genes (*1*,*4*). PRV can infect many kinds of mammals, including ruminants, carnivores, and rodents (*2*,*3*,*5*). However, pigs have been confirmed to be the primary hosts and reservoir of this virus (*6*–*8*). PRV infection is characterized by nervous system disorders and death in newborn piglets, respiratory disorders in older pigs, and reproductive failure in sows (*7*,*8*). Like other α herpesviruses, PRV infection can be a lifelong latent infection in the peripheral nervous systems of infected pigs, and these latently infected pigs can infect others under certain conditions (*7*–*9*). In this way, PRV causes devastating disease in pigs and economic losses worldwide.

Vaccination of pigs with attenuated live or inactivated vaccines is widely performed to reduce the huge economic losses caused by PRV infection (*10*–*12*). Although vaccination confers protection against disease, it does not prevent infection from a wild-type strain. Thus, both the virus in the vaccine and the super-virulent wild-type strain can establish latency within the same animal (*13*–*15*).

We report an outbreak of PRV infection that devastated the swine-producing regions of China in 2012. We systematically investigated the outbreak to identify the causative agent.

## The Study

**Figure 1:**
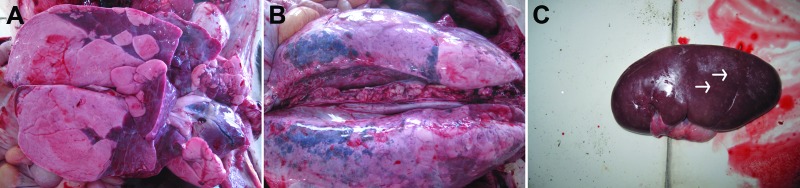
Necropsy specimens from pigs infected with pseudorabies virus. A) Pulmonary consolidation in the lung. B) Edema and hemorrhage of lung. C) Kidney with many yellow-white necrotic spots (arrows).

In January 2012, a previously unknown severe disease was observed in pigs on several farms in northern and eastern China. In Shandong Province, >80,000 pigs were infected. The affected pigs had high fever (>40.5°C), anorexia, coughing, respiratory distress, conjunctival serous and mucinous secretion, and posterior paralysis. The disease was first observed in older pigs and spread within 2–3 days to younger pigs. Duration of disease was 5–7 days. Rate of illness reached 50%, and mortality was 3%–5%. Most pig deaths were recorded on the third day after monitoring began. Abortion was observed in ≈35% of sows that were 70–90 days pregnant. Viscera (e.g., lung, kidney, heart, liver, and spleen) and serum samples were collected from dead pigs from different provinces. Pathologic examination showed the most striking gross lesions were consolidated in the lungs (Figure 1, panel A), with edema and hemorrhage (Figure 1, panel B). In addition, foci of yellow-white necrosis were observed in the kidneys of some dead pigs (Figure 1, panel C).

To gain insight into the etiologic agent of the disease, we conducted extensive and systematic diagnostic testing, including PCR, ELISA, viral isolation, immunohistochemical staining, and standard bacteriologic culture, to evaluate the specimens. Marc-145 cells, inoculated with various tissue homogenates, showed cytopathic effects. A specific PRV monoclonal antibody was used, and immunopositive cells were observed in infected tissue (Technical Appendix Figure 1, panels A, B). The PCR for inocula samples showed that many glycoprotein (g) genes of PRV could be amplified by using the primers specific to the unique gene fragments (Technical Appendix Figure 2). The PRV gE-ELISA assays (IDEXX Laboratories, Westbrook, ME, USA) indicated that serum samples from the sick pigs contained antibodies against wild-type, virulent PRV glycoprotein E but not against the vaccine strain (Table). All these results indicated that PRV was the causative agent of this disease. Our results also ruled out other suspected agents, such as classical swine fever, African swine fever, porcine reproductive and respiratory syndrome virus, and some bacterial infections. The 3 isolates found here are referred to as NVDC-PRV-BJ, NVDC-PRV-HEB, and NVDC-PRV-SD, according to the provinces from which they were isolated.

**Figure 2:**
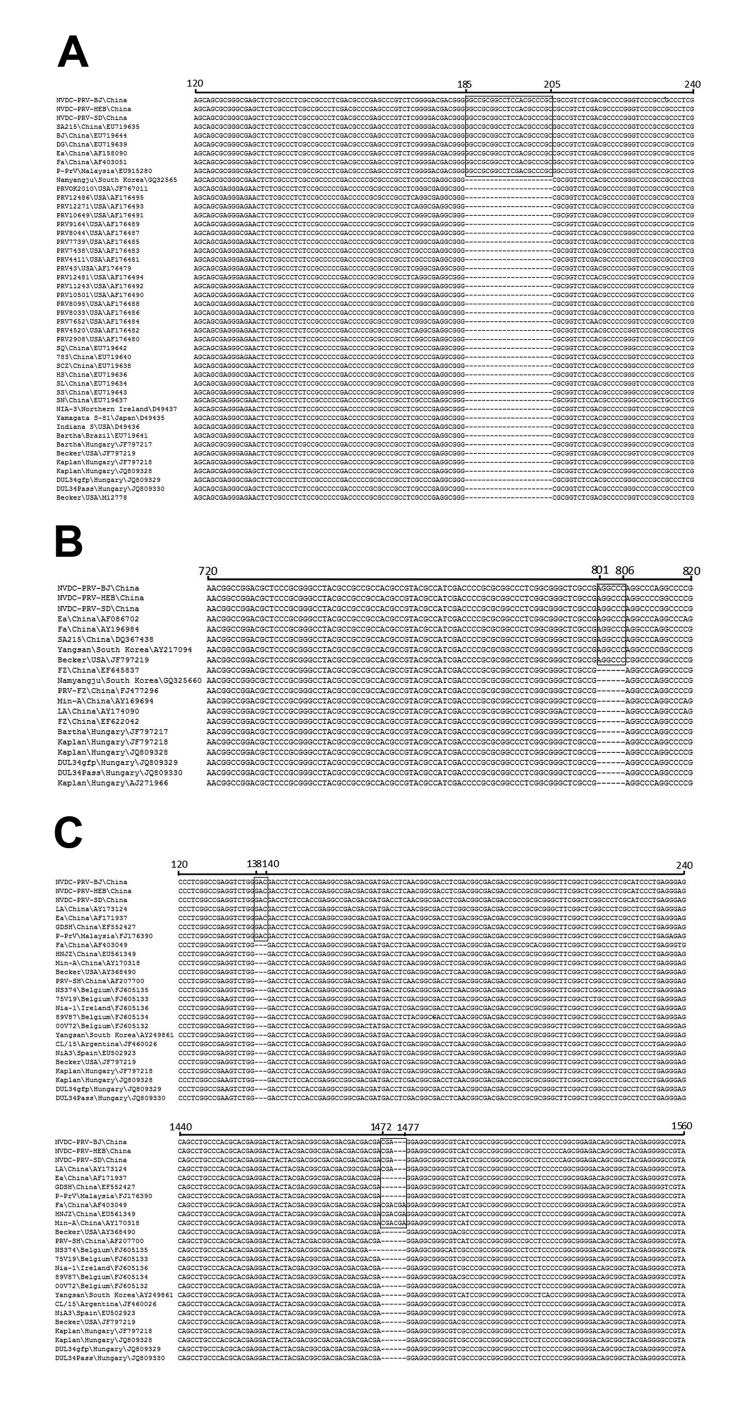
Alignment the partial sequences of glycoprotein (g) C (A), gD (B), and gE (C) genes of pseudorabies virus at the nucleotide level. Black box indicates the region of insertion.

**Table:** Antibodies to PRV gE in serum from PRV-infected pigs from different provinces, China, 2012

Sample origin province	Collection date	gE ELISA S/N ratio*
Shandong	2012 Jan	0.115
Beijing	2012 Feb	0.240
Hebei	2012 Feb	0.169
Tianjin	2012 Feb	0.171
Liaoning	2012 Mar	0.168
		0.274
		0.168
		0.173
		0.177
		0.179
		0.168
		0.158
		0.143

*S/N ratio was calculated as a ratio of the absorbance of a well with serum to the absorbance of a control well without serum. Serum with an S/N ratio of <0.60 was considered positive. PRV, pseudorabies virus; g, glycoprotein.

The pigs vaccinated with attenuated live PRV vaccines still showed clinical signs of PRV during the outbreak. To confirm the presence of PRV in these herds, 15 pigs were vaccinated with the current vaccine strain and then challenged with the NVDC-PRV-SD strain 21 days after vaccination. The results indicated that the vaccinated pigs had not been given completely effective protection against infection and exhibited obvious clinical signs of disease similar to the typical symptoms observed in the field, suggesting that the virulence of the newly isolated PRV strains had changed. This virulence may have caused the deaths of infected pigs.

To better understand the genetic relationship of the 3 PRV isolates found here to other PRV isolates, we amplified and sequenced the 15 major genes of the 3 isolates (Technical Appendix Table 1). Compared with other PRV isolates (Technical Appendix Table 2), there was a 21-nt insertion from nucleotide positions 185–205 in the gC gene, similar to SA215, BJ, DG, Ea, Fa, and P-PrV strains (Figure 2, panel A), which shared 100% nt identity with each other. The gD gene, like isolates Ea, Fa, SA215, and Yangsan, had 6 nt at positions 801–806 and shared 99.4%–99.8% identity with each other (Figure 2, panel B). There were 2 insertions of 6 discontinuous nucleotides each at positions 138–140 and 1472–1474 in the gE gene (Figure 2, panel C). These had 99.9%–100% identity with each other. The rest of the genes from the 3 isolates had no nucleotide insertions or deletion in common with the other PRV isolates. We further analyzed the relationship of these 3 isolates with other PRV isolates using a phylogenetic tree based on the gE gene; the 3 isolates formed a tightly clustered branch and were very closely related to other isolates from Asia (Technical Appendix Figure 3).

## Conclusions

We describe and analyzed a major outbreak of PRV in pigs in China. In these herds, all pigs had been vaccinated against PRV 3 times a year, at approximately the same time as each other. The disease spread to >6 provinces (including autonomous cities and regions) and caused considerable economic losses among local pig farms.

PRV has been recognized as a source of infection for pigs and continues to circulate globally among herds (*9*). In particular, pigs receiving attenuated live PRV vaccines showed typical clinical symptoms, which suggest that these isolates might have evolved new types of pathogenicity.

Even though the PRV isolates showed nucleotide insertions in the gC, gD, and gE genes, the molecular mechanisms underlying their high pathogenesis have yet to be elucidated. The origin of these lethal isolates within China is still obscure, although phylogenetic trees based on the gE gene here indicated that the 3 isolates are more closely related to the Asia PRV isolates, especially the China isolates, than to isolates from other countries. Because the virulence and origin of PRV is thought to be associated with multiple factors, whether such insertions are related to the virulence of PRV remains an issue and requires further investigation.

In summary, our study indicates that an outbreak of disease in pigs in China, which was of unprecedented scale, was caused by PRV infection. Other pathogens were ruled out. Our findings highlight the need to prevent and control the spread of this virus.

Technical Appendix Pseudorabies virus target genes and primers; sequences obtained from GenBank used in this study; immunohistochemical staining of lung and brain specimens of clinically sick pigs; PCR of infected tissues with specific primers for pseudorabies virus glycoprotein D–specific primers; and phylogenetic trees of glycoprotein E sequence.
