# Supplementary material for: Pathogenic Pseudorabies Virus, China, 2012
Source: Emerg Infect Dis. 2014 Jan;20(1):102–4. doi: 10.3201/eid2001.130531 (PMC3884716; doi:10.3201/eid2001.130531)
Supplement: Technical Appendix — Pseudorabies virus target genes and primers; sequences obtained from GenBank used in this study; immunohistochemical staining of lung and brain specimens of clinically sick pigs; PCR of infected tissues with specific primers for pseudorabies virus glycoprotein D–specific primers; and phylogenetic trees of glycoprotein E sequence. [file 13-0531-Techapp-s1.pdf]

Article DOI: http://dx.doi.org/10.3201/eid2001.130531

Pathogenic Pseudorabies Virus, China

Technical Appendix

Technical Appendix Table 1. PRV different target genes and primers in this study*

Reference PRV Kaplan strain target gene nucleotide length, bp	Primer sequences (5′→3′)	Amplicon, bp
gE	Forward primer (gE1F): ATGCGGCCCTTTCTGCTGCGCG	611
gD	Reverse primer (gE611R): ACAAAGAACACGGCCCGCGG	493
TK	Forward primer (gE592F): CCGCGGGCCGTGTTCTTTGT	568
gH	Reverse primer (gE1084R): CGTGGCCGTTGTGGGTCAT	634
gL	Forward primer (gE1066F): ATGACCCACAACGGCCACGTC	589
gM	Reverse primer (gE1734R): TTAAGCGGGGCGGGCATTCAACAGG	566
gN	Forward primer (gD1F): ATGCTGCTCGCAGCGCTATTGG	534
gB	Reverse primer (gD634R): TGAAGCTCTCGTCGTTCCAGC	658
gC	Forward primer (gD614F): GCTGGAACGACGAGAGCTTCA	474
gG	Reverse primer (gD1203R): CTACGGACCGGGCTGCGCTTTTA	773
gI	Forward primer (TK1F): ATGCGCATCCTCCGGATCTACCTC	471
gK	Reverse primer (TK566R): ACCAGCATGGCGTAGACGTTGC	574
PK	Forward primer (TK545F): GCAACGTCTACGCCATGCTGGT	628
11K	Reverse primer (TK1079R): TTTATTGGGATGACATACACATAGC	297
28K	Forward primer (gH1F): ATGCCCGCGTCGTCCGTGCGCCT	717
	Reverse primer (gH658R): AGCCGAGCGTGTACACGTGGAAG	732
	Forward primer (gH636F): CTTCCACGTGTACACGCTCGGCT	598
	Reverse primer (gH1310R): GAGAGGACGAGCAGGCTGAA	670
	Forward primer (gH1288F): TTCAGCCTGCTCGACGTCCTCTC	717
	Reverse primer (gH2061R): TCACGACCTGGCGTTTATTAACC	745
	Forward primer (gL1F): TTACTCGGCGGGGGGCCCTCCAT	728
	Reverse primer (gL471R): ATGTCGCCGCTCGTGGCGGTGCT	791
	Forward primer (gM1F): TTATTCAAAGCCGAGGTTCTCG	576
	Reverse primer (gM574R): CGTCGTGACGAACGTCGTCCT	547
	Forward primer (gM554F): AGGACGACGTTCGTCACGACG	939
	Reverse primer (gM1182R): ATGTGCGGGCCGCGCAACGCCGA	551
	Forward primer (gN1F): ATGGTCTCCTCCGCCGGGCTCT	666
	Reverse primer (gN297R): TTATACGCGCCGCCCCGCGG	297
	Forward primer (gB1F): CTACAGGGCGTCGGGGTCCTC	771
	Reverse primer (gB717R): GAGGACTACAACTACGTGCGCAT	
	Forward primer (gB695F): ATGCGCACGTAGTTGTAGTCCTC	
	Reverse primer (gB1427R): GCTACAACAGCACGCACGTGCT	

	Forward primer (gB1406F): AGCACGTGCGTGCTGTTGTAGC
	Reverse primer (gB2095R): GTGCGTCTCCAAGGCCGAGTACGT
	Forward primer (gB2072F): ACGTACTCGGCCTTGGAGACGCAC
	Reverse primer (gB2742R): ATGCCCGCTGGTGGCGGTCTTTG
	Forward primer (gC1F): ATGGCCTCGCTCGCGCGTGCG
	Reverse primer (gC717R): GAACTCGGGCTGGCTGTACAGGA
	Forward primer (gC695F): TCCTGTACAGCCAGCCCGAGTTC
	Reverse primer (gC1440R): TCACGGCCCCGCCCGGCGGTAGTA
	Forward primer (gG1F): ATGAAGTGGGCAACGTGGATC
	Reverse primer (gG728R): TCGTAGTAGTCGGCGTAGTCGTC
	Forward primer (gG706F): GACGACTACGCCGACTACTACGA
	Reverse primer (gG1497R): TCAGGCGGAGGCCACGTGGCGGT
	Forward primer (gI1F): ATGATGATGGTGGCGCGCGACGT
	Reverse primer (gI576R): CTGTCGTGCCACGATCCGACGA
	Forward primer (gI554F): TCGTCGGATCGTGGCACGACAG
	Reverse primer (gI1101R): TTATTGTTCCTCTGCGATGGTG
	Forward primer (gK1F): TCATCCAAATATGGTAATGTGCCG
	Reverse primer (gK939R): ATGCTCCTCGGCGGGCGCCCGCT
	Forward primer (PK1F): ATGTTGGCGATGTGGAGATGG
	Reverse primer (PK551 R): CTGTACAGATCGCACCGAAAGT
	Forward primer (PK530F): ACTTTCGGTGCGATCTGTACAG
	Reverse primer (PK1196R): TTATACGGTCCACATTCCAAAG
	Forward primer (11K1F): ATGGACACGTTCGACCCCAGC
	Reverse primer (11K297R): CTACACGTGCCGGGCGATGAT
	Forward primer (28K1F): ATGGGGGTGACGGCCATCACCGT
	Reverse primer (28K771R): CTAGGAGATGGTACATCGCG

*PRV, pseudorabies virus; g, glycoprotein.

Technical Appendix Table 2. PRV sequences obtained from GenBank used in this study*

Sequence coding for	Strain	GenBank accession no.	Origin
gC	Namyangju	GQ325659	South Korea
gD	PRV OK2010	JF767011	USA
gE	PRV12486	AF176495	USA
	PRV12271	AF176493	USA
	PRV10649	AF176491	USA
	PRV9164	AF176489	USA
	PRV8044	AF176487	USA
	PRV7739	AF176485	USA
	PRV7438	AF176483	USA
	PRV4411	AF176481	USA
	PRV43	AF176479	USA
	PRV12481	AF176494	USA
	PRV11243	AF176492	USA
	PRV10501	AF176490	USA
	PRV8095	AF176488	USA
	PRV8033	AF176486	USA
	PRV7652	AF176484	USA
	PRV4520	AF176482	USA
	PRV2908	AF176480	USA
	Ea	AF158090	China
	Fa	AF403051	China
	P-PrV	EU915280	Malaysia
	BJ	EU719644	China
	SQ	EU719642	China
	783	EU719640	China
	SCZ	EU719638	China
	HS	EU719636	China
	SL	EU719634	China
	SS	EU719643	China
	Bartha	EU719641	Brazil
	DG	EU719639	China
	SN	EU719637	China
	SA215	EU719635	China
	NIA3	D49437	Northern Ireland
	Yamagata S-81	D49435	Japan
	Indiana S	D49436	USA
	Bartha	JF797217	Hungary
	Kaplan	JF797218	Hungary
	Becker	JF797219	USA
	Kaplan	JQ809328	Hungary

Sequence coding for	Strain	GenBank accession no.	Origin
	DUL34 gfp	JQ809329	Hungary
	DUL34Pass	JQ809330	Hungary
	Becker	M12778	USA
	Namyangju	GQ325660	South Korea
	PRV-FZ	FJ477296	China
	Yangsan	AY217094	Korea
	Min-A	AY169694	China
	LA	AY174090	China
	FZ	EF645837	China
	FZ	EF622042	China
	Ea	AF086702	China
	Fa	AY196984	China
	SA215	DQ367438	China
	Bartha	JF797217	Hungary
	Kaplan	JF797218	Hungary
	Becker	JF797219	USA
	Kaplan	JQ809328	Hungary
	DUL34 gfp	JQ809329	Hungary
	DUL34Pass	JQ809330	Hungary
	Kaplan	AJ271966	Hungary
	Yangsan	AY249861	South Korea
	CL/15	JF460016	Argentina
	HNJZ	EU561349	China
	Becker	AY368490	USA
	PRV-SH	AF207700	China
	Ea	AF171937	China
	SA	GU262988	China
	NS374	FJ605135	Belgium
	Nia-1	FJ605136	Belgium
	89V87	FJ605134	Belgium
	00V72	FJ605132	Belgium
	75V19	FJ605139	Belgium
	LA	AY173124	China
	Fa	AF403049	China
	P-PrV	FJ176390	Malaysia
	NiA3	EU502923	Spain
	GDSH	EF552427	China
	Min-A	AY170318	China
	Kaplan	JF797218	Hungary
	Becker	JF797219	USA
	Kaplan	JQ809328	Hungary

Sequence coding for	Strain	GenBank accession no.	Origin
	DUL34 gfp	JQ809329	Hungary
	DUL34Pass	JQ809330	Hungary

*PRV, pseudorabies virus; g, glycoprotein.

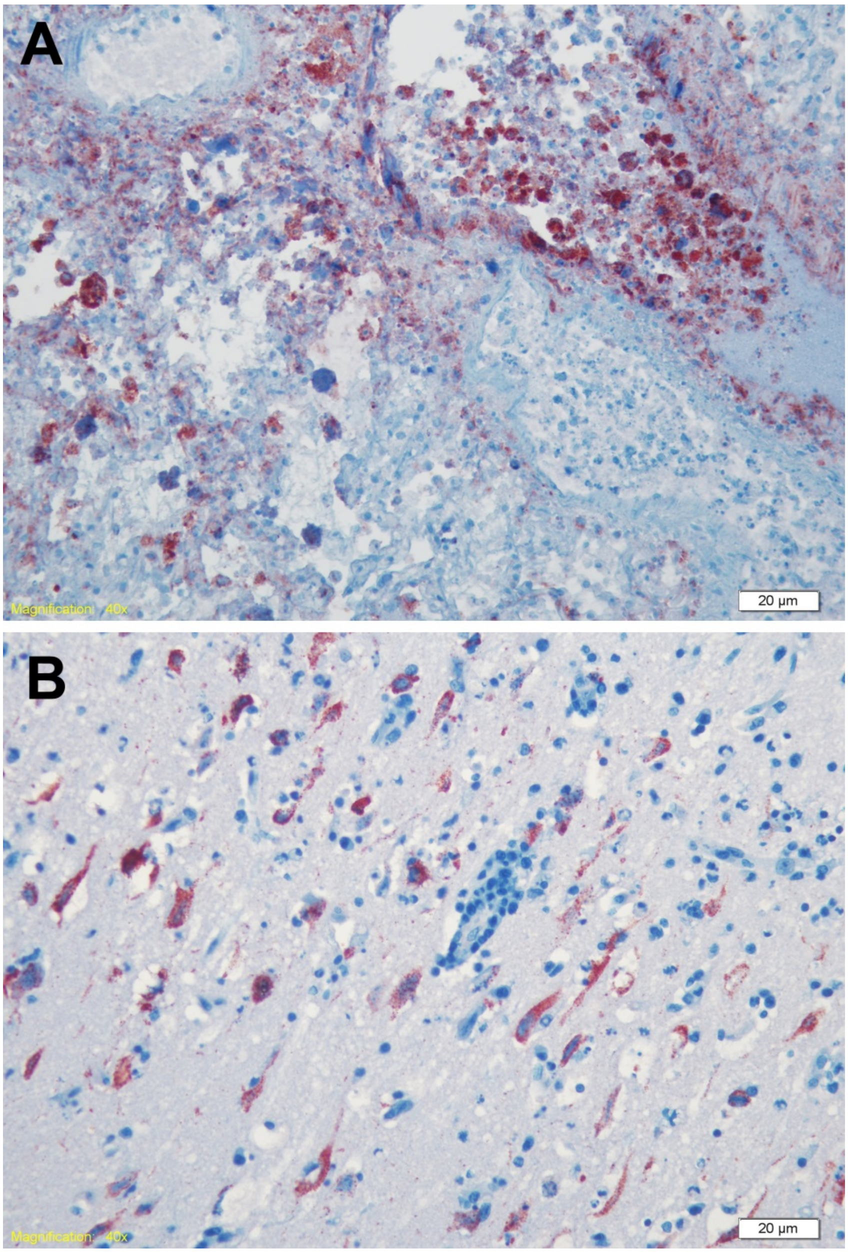

Technical Appendix Figure 1. Immunohistochemical staining of lung (A) and brain (B) specimens of clinically sick pigs. Original magnification ×100.

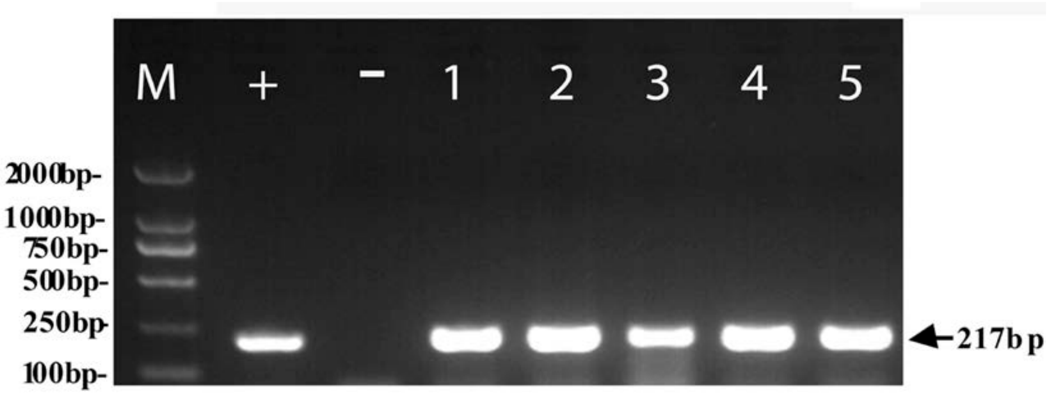

Technical Appendix Figure 2. PCR of infected tissues with specific primers for pseudorabies virus glycoprotein D (gD)–specific primers (forward primer 5′-CGGAGGACGAGCTGGGGCT-3′; reverse primer 5′-ACGTCCACGCCCCGCTTGAAGC-3′), with a fragment size of 217 bp. +: positive control; –, negative control; 1, 2, 3, 4, and 5: supernatants from the homogenized tissues of lungs, heart, tonsils, lymph nodes and serum, respectively. Arrow indicates the target fragment, 217 kb.

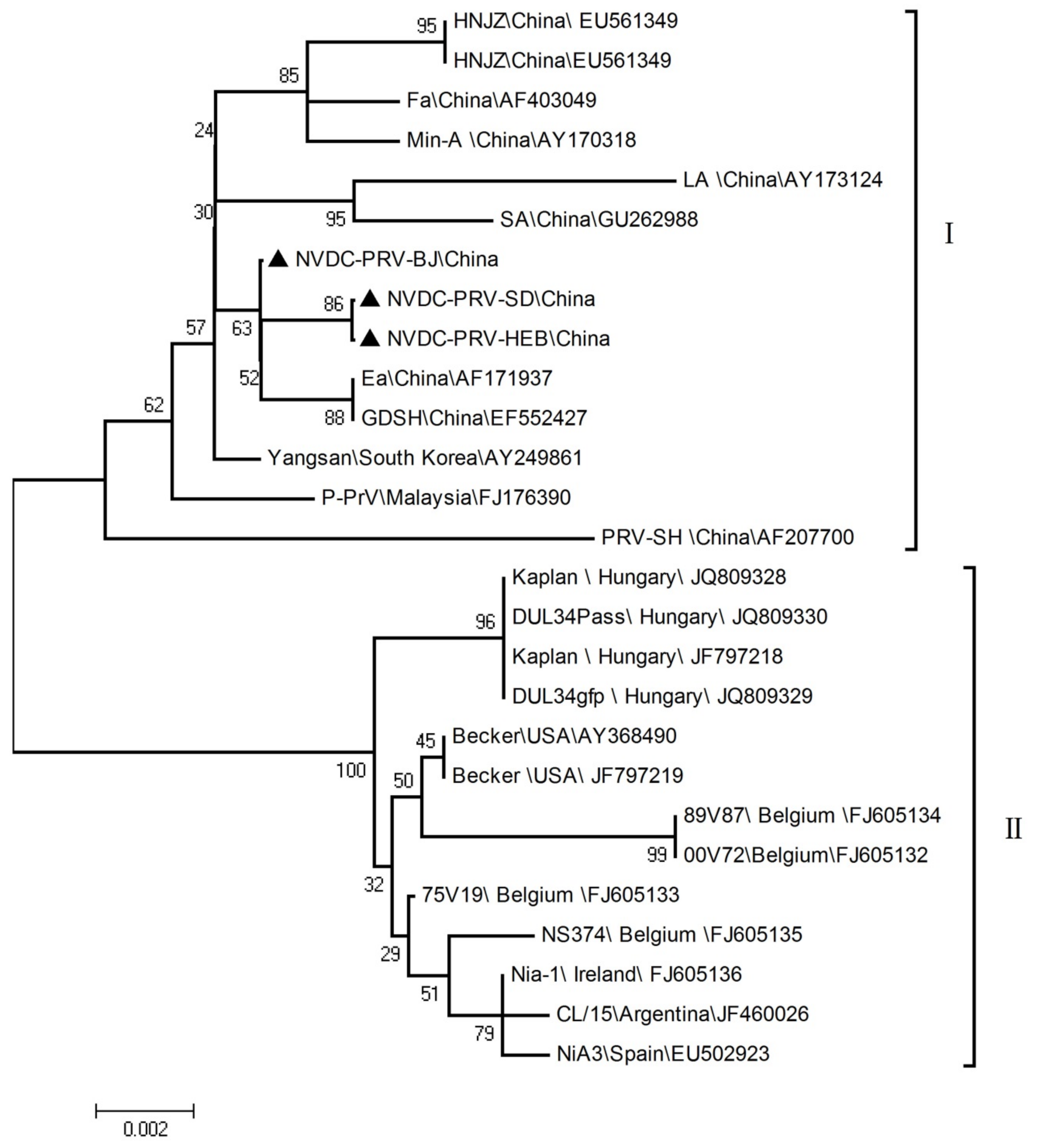

Technical Appendix Figure 3. Phylogenetic trees of the glycoprotein (g) E sequence. Unrooted trees constructed for gE sequences by using Mega v. 4.0 (www.megasoftware.net). The phylogenetic trees indicate the relationship of NVDC-PRV-BJ, NVDC-PRV-HEB, and NVDC-PRV-SD to other PRV isolates. These 3 isolates are indicated in the black triangle. Scale bar indicates number of substitutions per site.
